# Minding Impacting Events in a Model of Stochastic Variance

**DOI:** 10.1371/journal.pone.0018149

**Published:** 2011-03-31

**Authors:** Sílvio M. Duarte Queirós, Evaldo M. F. Curado, Fernando D. Nobre

**Affiliations:** 1 Centro de Física do Porto, Porto, Portugal; 2 Centro Brasileiro de Pesquisas Físicas and National Institute of Science and Technology for Complex Systems, Rio de Janeiro, Rio de Janeiro, Brazil; University of East Piedmont, Italy

## Abstract

We introduce a generalization of the well-known ARCH process, widely used for generating uncorrelated stochastic time series with long-term non-Gaussian distributions and long-lasting correlations in the (instantaneous) standard deviation exhibiting a clustering profile. Specifically, inspired by the fact that in a variety of systems impacting events are hardly forgot, we split the process into two different regimes: a first one for regular periods where the average volatility of the fluctuations within a certain period of time 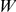 is below a certain threshold, 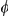, and another one when the local standard deviation outnumbers 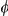. In the former situation we use standard rules for heteroscedastic processes whereas in the latter case the system starts recalling past values that surpassed the threshold. Our results show that for appropriate parameter values the model is able to provide fat tailed probability density functions and strong persistence of the instantaneous variance characterized by large values of the Hurst exponent (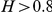), which are ubiquitous features in complex systems.

## Introduction

For the last years the physical community has broaden its subject goals to matters that some decades ago were too distant from the classical topics of Physics. Despite being apparently at odds with the standard motivations of Physics, this new trend has given an invaluable contribution toward a more connected way of making Science, thus leading to a better understanding of the world surrounding us [1]. Within this context, the major contribution of physicists is perhaps the quantitative procedure, reminiscent of experimental physics, in which a model is proposed after a series of studies that pave the way to a reliable theory. This path has resulted in a series of findings which have helped such diverse fields as physiology, sociology and economics, among many others [2]–[4]. Along these findings, one can mention the determination of non-Gaussian distributions and long-lasting (power-law like) correlations [5]–[7]. Actually, by changing the observable, the conjunction of the two previous empirical verifications is quite omnipresent. For this reason and regardless the realm of the problem very similar models have been applied with particular notoriety to discrete stochastic processes of time-dependent variance based on autoregressive conditional heteroscedastic models [8]. That is to say, most of these models are devised taking basically into account the general features one aims at reproducing, rather than putting in elements that represent the idiosyncracies of the system one is surveying. For instance, many of the proposals cast aside the cognitive essence prevailing on many of these systems, when it is well known that in real situations this represents a key element of the process [9]. On the other hand, intending to describe long-lasting correlations, long-lasting memories are usually introduced thus neglecting the fact that we do not traditionally keep in mind every happening. As a simple example, we are skilled at remembering quotidian events for some period. However, we will discard that information as time goes by, unless the specific deed either created an impact on us or has to do with something that has really touched us somehow. In this case, it is likely that the fact will be remembered forever and called back in similar or related conditions, which many times lead to a collective memory effect [10].

In this work, we make use of the celebrated heteroscedastic model, the 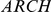 process [11] and modify it by pitching at accommodating cognitive traits that lead to different behavior for periods of high agitation or impact. Particularly, we want to stress on the fact that people tend to recall important periods, no matter when they took place. To that end, we introduce a measure of the local volatility, as well as a volatility threshold, so that the system changes from a normal dynamics, in which it uses the previous values of the variable to determine its next value, to a situation in which it recalls the past and compares the current state with previous states of high volatility, even if this past is far.

### Standard models of heteroscedasticity

The Engle's formulation of an autoregressive conditional heteroscedastic (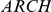) time series [11] represents one of the simplest and effectual models in Economics and Finance, for which he was laureated the Nobel Memorial Prize in Economical Sciences in 2003 [12]. Explicitly, the 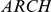 corresponds to a discrete time, 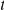, process associated with a variable, 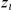,
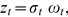
(1)with 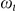 being an independent and identically distributed random variable with zero mean and standard deviation equal to one. The quantity 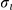 represents the time-dependent standard deviation, which we will henceforth name *instantaneous volatility* for mere historical reasons. Traditionally, a Gaussian is assigned to the random variable 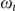, but other distributions, namely the truncated 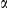-stable Lévy distribution and the 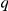-Gaussian (Student-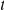) have been successfully introduced as well [13], [14]. In his seminal paper, Engle suggested that the values of 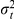 could be obtained from a linear function of past squared values of 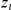,
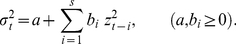
(2)In financial practice, *viz.,* price fluctuations modelling, the case 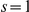 (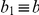) represents the very most studied and applied of all the 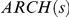-like processes. The model has been often applied in cases where it is assumed that the variance of the observable (or its fluctuation) is a function of the magnitudes of the previous occurrences. In a financial perspective, Engle's proposal has been associated with the relation between the market activity and the deviations from the normal level of volatility 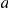, and the previous price fluctuations making use of the impact function [8]. Alternatively, recent studies convey the thesis that leverage can be responsible for the volatility clustering and fat tails in finance [15]. Nonetheless, the heteroscedastic 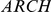-like processes has been repeatedly used as a forecasting method. In other words, one makes use of the magnitude of previous events in order to indicate (or at least to bound) the upcoming event (see e.g. [16], [17]). In respect of its statistical features, although the time series is completely uncorrelated, 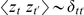, it can be easily verified that the covariance 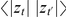 is not proportional to 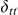. As a matter of fact, for 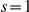, it is provable that 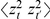 decays according to an exponential law with a characteristic time 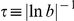. This dependence does not reproduce most of the empirical evidences, particularly those bearing on price fluctuations studies. In addition, the introduction of a large value of 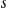 used to give rise to implementation problems [18]. Expressly, large values of 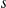 augment the difficulty of finding the appropriate set of parameters 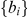 for the problem under study as it corresponds to the evaluation of a large number of fitting parameters. Aiming to solve this short-coming of the original 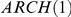 process, the 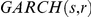 process was introduced [19] (where 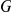 stands for generalized), with Eq. (2) being replaced by,
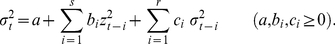
(3)In spite of the fact that the condition, 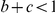, guarantees that the 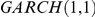 process exactly corresponds to an infinite-order 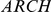 process, an exponential decay for 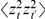, with 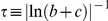 is found.

Although the instantaneous volatility is time dependent, the 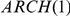 process is actually stationary with the *stationary variance* given by,
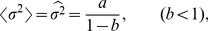
(4)(herein 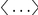 represents averages over samples at a specified time and 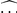 denotes averages over time in a single sample). Moreover, it presents a stationary probability density function (PDF), 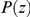, with a kurtosis larger than the kurtosis of distribution 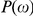. Namely, the fourth-order moment is,
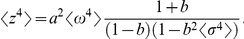
This kurtosis excess is precisely the outcome of the dependence of 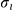 on the time (through 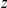). Correspondingly, when 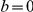, the process is reduced to generating a signal with the same PDF of 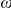, but with a standard variation equal to 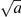. At this point, it is convenient to say that, for the time being and despite several efforts, there are only analytical expressions describing the tail behavior of 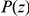 or the continuous-time approximation of the 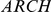
(1) process with the full analytical formula still unknown [14], [20].

In order to cope with the long-lasting correlations and other features such as the asymmetry of the distribution and the leverage effect, different versions of the 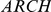 process have been proposed [8], [18]. To the best of our knowledge, every of them solve the issue of the long-lasting correlations of the volatility by way of introducing an eternal dependence on 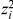 in Eq. (2), 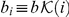, with 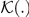 representing a slowly decaying function [8], [21]. Most of these generalizations can be encompassed within the fractionally integrated class of 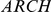 processes, the 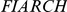 [22]–[25]. The idea supporting the introduction of a power-law for the functional form of 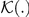 is generally based on the assumption that the agents in the market make use of exponential functions 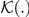 with a broad distribution of relaxation times related to different investment horizons [26], [27]. This type of model has achieved a huge popularity in the replication of non-Gaussian time series in several areas, such as biomedicine, climate, engineering, and physics (a few examples can be found in [28]–[35]).

As described above, the statistical features of the macroscopic observables are the result of the nature of the interactions between the microscopic elements of the system and the relation between microscopic as well as the macroscopic observables. In the case of the “financial” 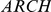 process, it was held that 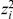 bears upon the impact of the price fluctuations on the trading activity. On the one hand, it is understood that the impact of the price fluctuations (or trading activity) on the volatility does not merely come from recent price fluctuations and it does actually involve past price fluctuations. In finance, upgraded versions of heteroscedasticity models use multi-scaling, *i.e.*, it is assumed that the price will evolve by modulating the volatility according to the volatility over different scales (days, weeks, months, years, etc.) [36] in order to smooth their possible misjudgement about the volatility. However, in practice, these models do not differ much from 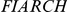-like proposals at the level of the results we are pointing at. Alternately, it is worthwhile to look upon the 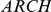 proposal as a mechanism of forecast [16], [17]. In this way, the simplest approach, the 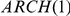, represents an attempt to foresee future values just taking into account recent observations, whereas models like the 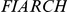 bear in mind all the history weighting each past-value according to some kernel functional.

### Minding impacting events

In our case, we want to emphasize the fact that people tend to recall periods of high volatility (*i.e.*, impact) in the system, no matter when they took place, by changing the surrounding conditions as agent-based models suggested [37], [38]. Hence, we introduce a measure of the local volatility,
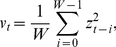
(5)and a threshold, 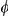, so that instead of Eq. (2), the updating of 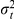 goes as follows:
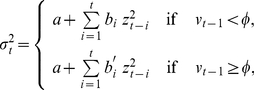
(6)where 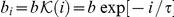 [39], [40]. Therefore, if we assume the financial market perspective, we are implicitly presuming that the characteristic time, 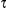, is Dirac delta or at least narrow distributed, so that the exponential functional is a valid approximation. This approach is confirmed by recent heuristic studies in which it has been verified that the largest stake of the market capitalization is managed by a small number of companies that apply very similar strategies [41]. With the second branch equation we intend to highlight the difference in behavior of the "normal" periods of trading and the periods of significant volatility, in which the future depends on the spells of significant volatility in the past as well. The values 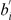 are defined as,
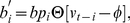
(7)with 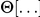 being the Heaviside function and 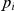 is a factor that represents a measure of the similarity (in the volatility space) between the windows of size 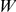 with upper limits at 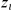 and 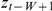, respectively. Analytically, this is equivalent to mapping segments in the form 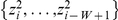 into vectors in 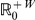 and afterward computing a normalized internal product-like weight,
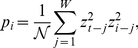
(8)where, for the sake of simplicity, we set aside the time dependence of 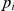 and 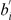 in the equations, while 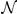 represents the normalization factor such that 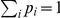 for all 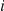 (with fixed 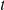).

We are therefore dealing with a model characterized by 5 parameters, namely: 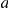 (the normal level of volatility) and 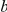 (the impact of the observable in the volatility), which were both first introduced by Engle in [11]; 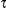, put forward in exponential models; and two new parameters 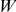 (representing the volatility spell) and 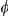 that we will reduce to a single extra parameter. If we think of trading activities, our proposal introduces a key parameter, the volatility threshold, 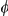, which signals a change in behavior of the agents in the market. At present, significant stake of the trading in financial markets is dominated by short-term positions and thus a good part of the dynamics of price fluctuations can be described by Eq. (2), or by functions with an exponential kernel. As soon as the market fluctuates excessively, *i.e.*, the volatility soars beyond the threshold, the market changes its trading dynamics. The main forecast references are obviously the periods where the volatility has reached high levels and afterward, the periods of those which are most similar; this is the rationale described by our Eq. (8). Thence, our proposal is nothing but the use of simple mechanisms that in a coarse-grained way master a good part of our decisions.

## Results

### General results

In this section we present the results obtained by the numerical implementation of the model. For comparison, we will use the results of a prior model that can be enclosed in the class of 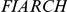 processes [25]. There, the adjustment of the parameters comes from the delicate balance between the parameter 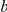, which is responsible for introducing deviations of the volatility from its normal level 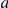, and the parameter controlling the memory. On the one hand, large memory has the inconvenient effect of turning constant the instantaneous volatility, so that after a seemly number of time steps the value of 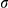 becomes constant, hence leading to a Gaussian (or close to it) distribution of the variable 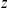, independently of how large 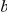 is. On the other hand, short memory is unable to introduce long-range correlations in the volatility, although it enhances larger values of kurtosis excess. The model we introduce herein is rather more complex. In order to deal with the change of regime, we define a parameter establishing this alteration and we need to specify 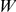 and 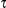. Henceforth, we have assumed 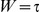, which is very reasonable as it imposes that the volatility and the time scale that the agents in the market use to assess the evolution of the observable are the same. In order to speed up our numerical implementation, we have imposed a cut-off of 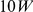 in the computation of the first line in Eq. (6). This approximation turns the numerical procedure much lighter with a negligible effect because the influence of the discarded past is not much relevant in numerical terms (within standard numerical implementation error). In all of our realizations, we have used a normalized level of expected volatility, 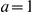, and we have defined the volatility threshold in units of 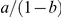, following a stationary approach, as well.

We have adjusted the probability distributions of 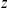 by means of the distribution,
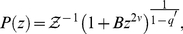
(9)the behavior of which follows a power-law distribution for large 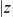 with an exponent equal to 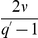 and where (using Ref. [42], sec. 3.194),
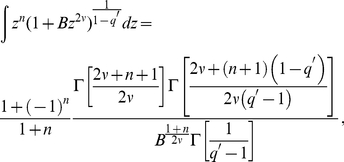
(10)

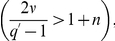
 and 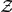 represents the previous integral with 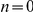. The fittings for the probability density distribution (9) were obtained using non-linear and maximum log-likelihood numerical procedures and the tail exponents double-checked with the value given by the Hill estimator [43], [44]. As a matter of fact, values of 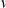 different from 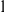 have only been perceived for large values of 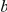 and small values of 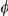 (slightly larger) or large values of 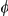 (slightly smaller). For 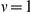 and 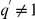, the PDF corresponds to a 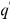-Gaussian distribution (or Student-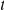 distribution) [45] and when 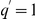 we have either the Gaussian (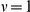) or the stretched distribution (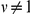). Since that in the majority of the applications one is interested in the tail behavior, we have opted for following the same approach by defining the tail index as,
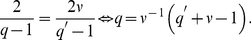
(11)In spite of the fact that other functional forms could have been used, we have decided on Eq. (9) because of its statistical relevance and simplicity (in comparison with other candidates involving special functions, namely the hypergeometric). Moreover, the 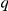-Gaussian (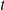-Student) is intimately associated with the long-term distribution of heteroscedastic variables since it results in the exact distribution when the volatility follows an inverse-Gamma distribution [35], [46]–[48].

Concerning the persistence of the volatility, we have settled on the Detrended Fluctuation Analysis (DFA) [49], which describes the scaling of a fluctuation function related to the average aggregated variance over segments of a time series of size 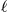,
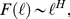
(12)where 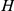 is the Hurst exponent. Although it has been shown that Fluctuation Analysis methods can introduce meaningful errors in the Lévy regime [50], we have verified that for our case, which stands within the finite second-order moment domain, the results of DFA are so reliable as other scaling methods.

**Figure 1:**
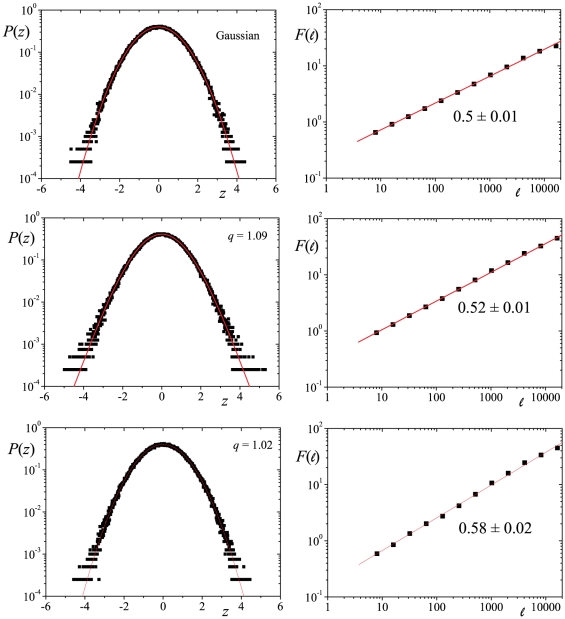
Probability density functions 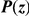 vs 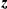 in a log-linear scale on the left column; On the right column the fluctuation functions 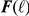 vs 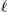 for 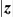 in a log-log scale. The values of the model parameters are: 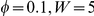 yielding 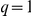 and 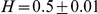 (upper panels); 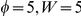 yielding 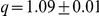 and 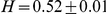 (middle panels); 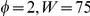 yielding 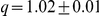 and 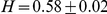 (lower panels). The results have been obtained from series of 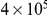 elements and the numerical adjustment of 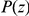 gave values of 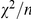 never greater than 0.00003, with 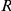 never smaller than 0.998.

Let us now present our results for 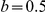, which is able to depict the qualitative behavior of the model for small 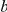. This case corresponds to a situation of little deviation from the Gaussian, when long-range memory is considered. In accordance, we can analyse the influence of the threshold 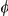 and 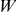. Overall, we verify a very sparse deviation from the Gaussian. Keeping 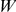 fixed and varying 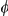, we understand that for small values of 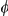 the distribution of 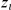 is Gaussian and the Hurst exponent of 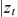 is 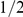. It is not hard to grasp this observation if we take into account that, by using small values of 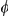, we are basically employing almost all of the past values which limits the values of instantaneous volatility to a constant value after a transient time. As we increase the value of 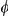, we let the dynamics be more flexible and therefore the volatility is able to fluctuate, resulting in a kurtosis excess. For small values of 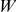, the Hurst exponent is slenderly different from 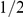 and the value of the Hurst exponent increases with 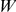. However, because of the small value of 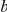, the rise of 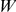 turns out the distribution of 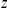 barely undistinguishable from a Gaussian. This behavior is described in Fig. 1. We have obtained a Gaussian distribution and a Hurst exponent 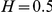 for small values of 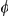 (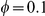) and 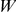 (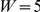). When we augment the value of the threshold, 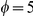, the system is loose and the instantaneous volatility is able to fluctuate leading to the emergence of tails (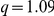) and a subtle increase of the Hurst exponent (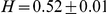). Hiking up both 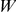 and 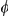 (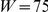 and 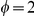), we have achieved large values of the Hurst exponent (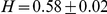), but the small value of 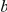 is not sufficient to induce relevant fluctuations, bringing on a distribution that is almost Gaussian (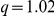). The distribution fittings were assessed by computing the critical value 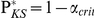 from the Kolmogorov-Smirnov test [51] that are equal to 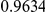 and 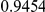, respectively.

**Figure 2:**
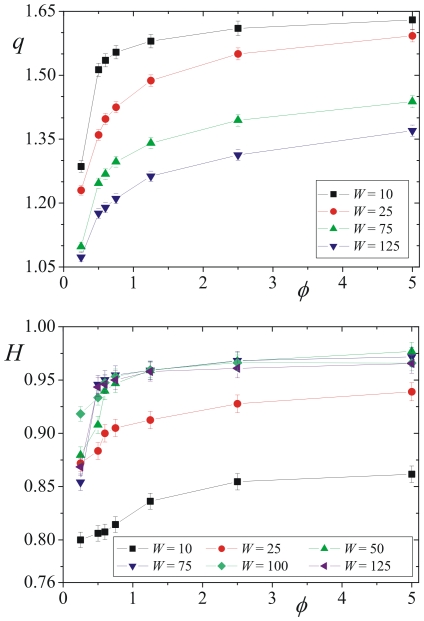
Value of the tail index 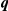 vs parameter 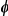 for several values of 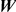 and 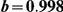 according to the adjustment procedures mentioned in the text in the upper panel. In the lower panel Hurst exponent 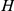 vs 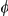. The results have been obtained from series of 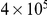 elements and the numerical adjustment of 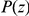 gave values of 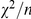 never greater than 0.00003 with 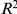 never smaller than 0.998. Regarding the values of the Hurst exponent, the absolute error has never been greater that 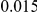 and a linear coefficient 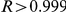.

As we increase the value of 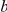, we favor the contribution of the past values of the price dynamics, thus, for the same value of 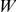 we are capable of achieving larger values of the kurtosis excess, that we represent by means of the increase of the 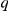 index. The same occurs for the Hurst exponent. This general scenery is illustrated in Fig. 2 for the value 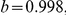 where we present the dependence of 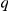 and 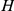 with 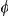, for different choices of 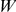. Again, the higher 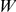, the lower the tail index 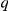, because the extension of the memory surges a weakening of the fluctuations in the volatility. The opposite occurs with the Hurst exponent, which increases towards unit (ballistic regime) as we consider 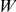 larger, for obvious reasons. In all the cases of 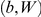 investigated, we verified that both 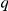 and 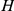 augment with 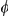. The assessment of the numerical adjustments is provided in Tab. 1 in the form of the 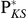 critical values from the Kolmogorov-Smirnov test [51]. The only case we obtained a value 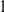 (within a five-digit precision) was for the pair 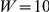 and 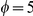, which results in a value quite close to the limit of finite second-order moment (a fat-tailed distribution with 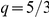). At this point it is worth saying that we have investigated the likelihood of other well-known continuous distributions, such as the stretched-exponential, the simple 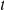-Student, Lévy, and Gaussian. Nonetheless, the fittings carried with Eq. (9) outperformed every other analyzed distribution.

**Figure 3:**
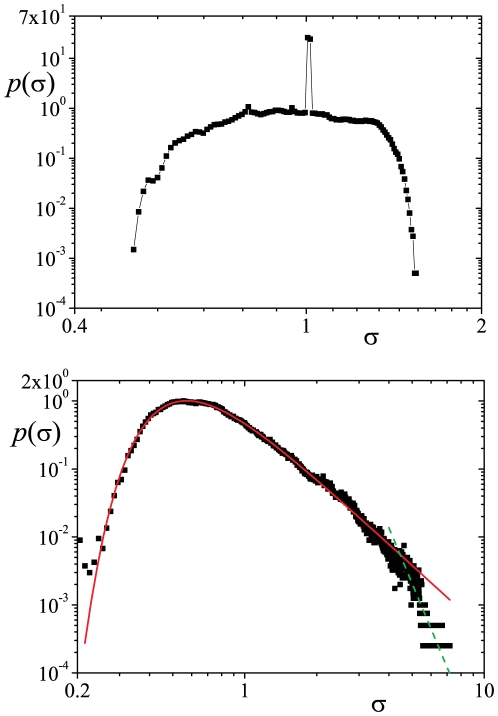
Probability density function of the instantaneous volatility 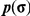 vs 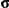 for two different 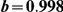. In the upper panel: 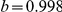, 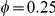 and 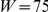 which leads to a sharply peaked distribution around 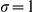 and to a 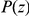 tail index 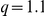. In the lower panel: 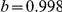, 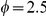 and 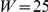 that results in a broader distribution largely described by a type-2 Gumbel distribution with 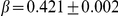 and 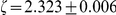 (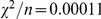 and 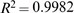). For 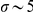, 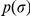 changes its behavior to a faster decay with an exponent equal to 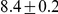 represented by the gray symbols. The ANOVA test of the type-2 Gumbel adjustment (up to 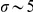) have yielded a sum of squares of 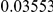 (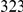 degrees of freedom) and 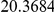 (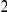 degrees of freedom) for the error and the model, respectively. The uncorrected value of the sum of squares is 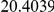 (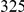 degrees of freedom) and the corrected total is 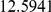 (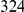 degrees of freedom). The empirical distribution function has been obtained from series of 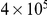 elements.

**Figure 4:**
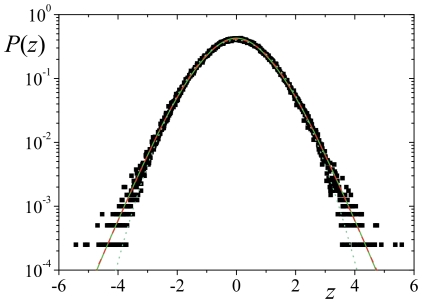
Probability density function 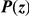 vs 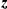. The points represent the empirical distribution function for 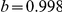, 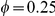 and 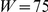; the dashed red line is our adjustment with Eq. (9) with 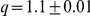, 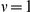 and 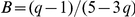 [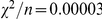 and 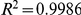]; the green line is PDF (15) with 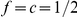 and the dotted cyan line is the Normal distribution.

Concerning the instantaneous volatility, 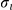, we verified that the Dirac delta distribution, 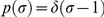, starts misshaping and short tails appear as we depict in Fig. 3 (upper panel) for the case 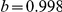, 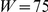 and 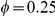. Considering this particular case, we can present relevant evidence of the effectiveness of our proposed probability distribution approach. The empirical distribution function in the upper panel of Fig. 3 may be simply approximated by
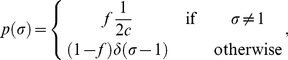
(13)with 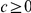, 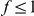, and 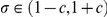; when 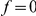 we recover the homoscedastic process distribution as a particular case. Reminding that at each time step the distribution is a Gaussian (conditioned to a time-dependent value of 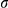) the long-term distribution is,
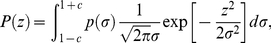
(14)which gives (Ref. [42], sec. 3.351),
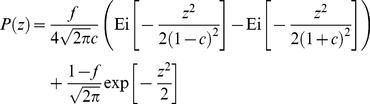
(15)where 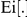 is the Exponential Integral function (see e.g. Ref. [52]). Considering 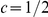 (which is appropriate to the case shown) and taking for the sake of simplicity 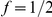, we obtain the function presented in Fig. 4, the kurtosis of which is 
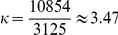
 (making use of Ref. [42], sec. 5.221). Actually, this curve is represented in the scaled variable 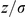 so that the standard deviation, which is originally equal to 
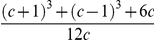
, becomes equal to one, like in other depicted distributions. The accordance between this distribution and the empirical distribution is quite remarkable since it emerges from no numerical adjustment and can be further improved by tuning the values of 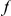 and 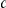. Regardless, this kurtosis value is only 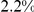 larger than our numerical adjustment (see Table 1 for the goodfness of fitting). Furthermore, comparing the distributions by means of the symmetrized Kullback-Leibler divergence 
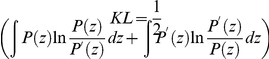
, we obtain a value of 0.00014 that is 19 times smaller than the distance between our fitting and a Gaussian. These results show that the PDF of Eq. (9) not only provides a good description of the data, but it is much more manageable as well.

**Table 1:** Critical values 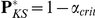 from the Kolmogorov-Smirnov test for typical pairs 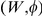 used for adjustments.

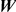	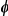	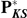	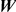	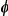	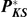
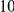	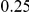	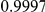	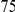	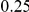	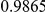
	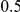	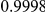		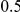	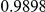
	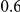	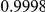		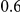	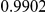
	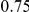	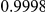		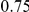	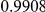
	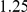	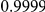		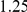	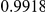
	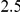	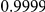		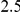	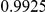
	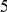	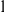		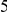	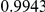
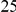	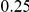	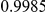	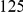	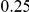	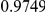
	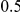	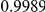		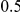	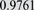
	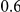	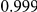		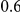	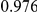
	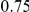	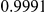		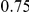	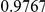
	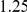	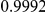		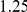	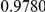
	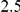	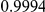		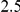	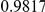
	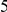	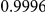		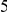	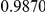

Cases for which the kurtosis excess is relevant (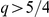) stem from wider distributions of 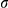 (see the lower panel of Fig. 3). Actually, it is the emergence of larger values of the instantaneous volatility that brings forth fat tails. Although we have not been successful in describing the whole distribution, we have verified that, for values of 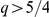, the distribution 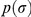 is very well described by a type-2 Gumbel distribution,
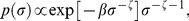
(16)and after certain value of 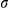 the distribution sharply decreases according to a power-law with a large exponent. We credit this sheer fall to the threshold 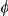, which introduces a sharp change in the dynamical regime of the volatility and thus in its statistics. In finance, such a cut-off is more than plausible as real markets do suspend trading when large price fluctuations occur. This also grants feasibility to descriptions based on truncated power-law distributions [6]. Moreover, a fall off is also presented in the quantity 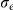 of Fig. 3 in Ref. [53]. It is known that for heteroscedastic models the tail behavior of the long-term distribution is governed by the asymptotic limit of 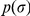 when 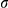 tends to infinity. For the case of distribution (16), this limit is the power-law 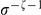 and therefore we can verify that the asymptotic behavior of the long-term distribution of the variable 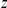,
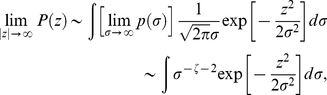
(17)yields a power-law distribution (applying Ref. [42], sec. 3.326),
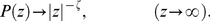
(18)For 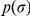 following an exponential decay in the form exp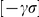, a similar procedure yields,
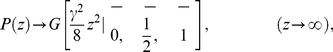
(19)where 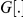 is the Meijer G-function [42], [52]. It is worth noting that in an effort to obtain a full description of 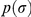 we also used a function such as 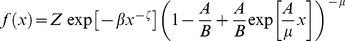 which allows the appearance of a crossover from a power law to an exponential decay. Nonetheless, it did not provide better results.

It is worth saying that we can reduce the number of parameters to 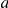, 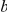 and 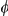, *i.e.*, apply the simple 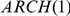 process, and obtain fat tails and persistence still.

### Comparison with a real system

**Figure 5:**
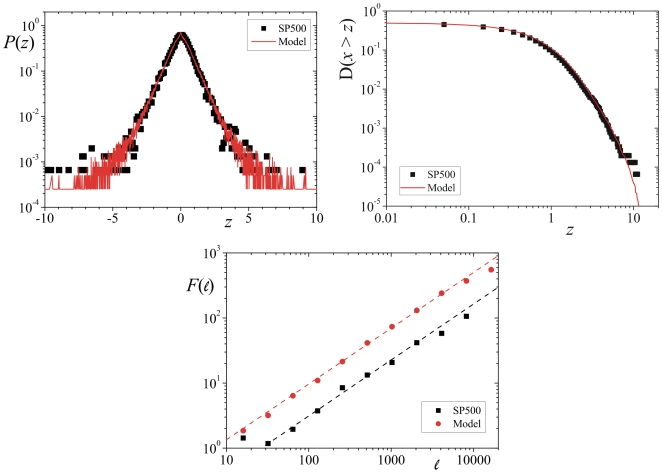
Probability density function 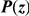 vs 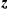. In the upper panels and on the left side we have 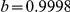, 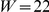 and 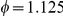 (full line) [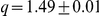 with 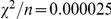 and 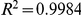] and the 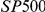 daily log-index fluctuations (symbols) [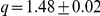 with 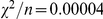 and 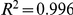] in the log-linear scale and on the right side the complementary cumulative distribution function 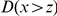 vs 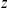 for case shown on the left. Lower panel: Fluctuation function 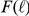 vs 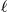 for the same parameters above [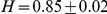, with 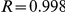] (red circles) and for the 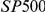 daily log-index fluctuations [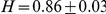, with 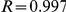] (black squares) in a log-log scale.

Following this picture, we can now look for a set of parameters that enable us to replicate a historic series such as the daily (adjusted) log-index fluctuations, 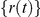, of the SP500 stock index, 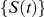, between 3rd January 1950 and 12th April 2010 (14380 data points) with,
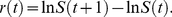
(20)The adjusted values of the index take into account dividend payments and splits occurred in a particular day. Inspecting over a grid of values of 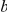, 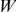 and 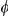, we have noted that the values of 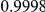, 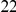 and 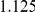, respectively, yield values of 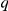 and 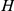 for 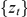 that are in good agreement with a prior analysis of 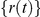 which gave 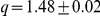 (using a simple 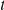-Student distribution) and 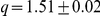 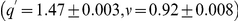 [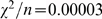, 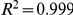 and 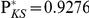](using the PDF of Eq. (9)) and persistence exponent 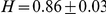 (see Fig. 4). Comparing the numerical distribution of our model with the data we obtained 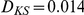 and a 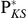 critical value equal to 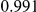 from the two-sample Kolmogorov-Smirnov test [51], while the comparison between the distribution of the numerical procedure and the adjustment of the SP500 empirical distribution function yielded 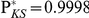. Once again we have tested other possible numerical adjustments and the only other relevant distribution was the stretched exponential with 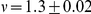 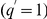 which has given a 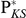 different from 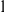 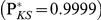, but a significantly larger value of 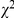 [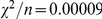, 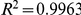] (see Fig. 5).

It is worthy to be mentioned that all the three values of the parameters are plausible. First, within an application context, 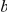 is traditionally a value robustly greater than 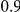. Second, 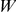 is close to the number of business days in a month and last, but not least, 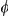 is somewhat above the average level of the mean variance presented above. This provides us with a very interesting picture of the dynamics. Specifically, at a relevant approximation we can describe this particular system as monitoring the magnitude of its past fluctuations with a characteristic scale of a month, from which it computes the level of impact resulting in an excess of volatility. Actually one month moving averages are established indicators in quantitative analyses of financial markets. When the volatility in a period of the same order of magnitude of 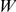 surpasses the value 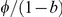, then the system recalls previous periods of time, no matter how long they happened, in which a significant level of volatility excess occurred. Those periods are then averaged in order to determine the level of instantaneous volatility 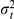.

## Discussion

We have studied a generalization of the well-known 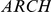 process born in a financial context. Our proposal differs from other generalizations, since it adds to heteroscedastic dynamics the ability to reproduce systems where cognitive traits exist or systems showing typical cut-off limiting values. In the former case, when present circumstances are close to extreme and impacting events, the dynamics switches to the memory of abnormal events. By poring over the set of parameters of the problem, namely the impact of past values, 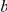, the memory scale, 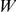, and the volatility threshold, 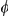, we have verified that we are able to obtain times series showing fat tails for the probability density function and strong persistence for the magnitudes of the stochastic variable (directly related to the instantaneous volatility), as it happens in several processes studied within the context of complexity. In order to describe the usefulness of our model we have applied it to mimic the fluctuations of the stock index 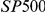, we verified that the best values reproducing the features of its time series are 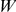 close to one business month and 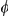 greater that the mean variance of the process which is much larger than the normal level of volatility for which trading is not taken into account. Concerning the volatility, we have noticed that for the problems of interest (*i.e.*, fat tails and strong persistence), the distributions are very well described by a type-2 Gumbel distribution in large part of the domain, which explains the emergence of the tails.

## Materials and Methods

Our results have been obtained from numerical simulation using code written in fortran language and run on the 64-bit ssolarII cluster (http://mesonpi.cat.cbpf.br/ssolar/).
